# Uncomfortable realities: the challenge of creating real change in Europe’s consolidating hospital sector

**DOI:** 10.1186/s12913-016-1389-3

**Published:** 2016-05-24

**Authors:** Patrick Jeurissen, Antonio Duran, Richard B. Saltman

**Affiliations:** Radboud University Medical Center Nijmegen, Nijmegen, The Netherlands; Ministry of Health, Welfare, and Sports, The Hague, The Netherlands; CEO AIIDMHealthSeville, Sevilla, Spain; Department of Health Policy and Management, Rollins School of Public Health, Emory University, Atlanta, GA USA

**Keywords:** European hospitals, Governance, Privatization, Payment reform, Cost-containment, Hospital management, Hospital business models

## Abstract

**Background:**

This article examines uncomfortable realities that the European hospital sector currently faces and the potential impact of wide-spread rationalization policies such as (hospital) payment reform and privatization.

**Methods:**

Review of relevant international literature.

**Results:**

Based on the evidence we present, rationalization policies such as (hospital) payment reform and privatization will probably fall short in delivering better quality of care and lower growth in health expenses. Reasons can be sought in a mix of evidence on the effectiveness of these rationalization policies. Nevertheless, pressures for different business models will gradually continue to increase and it seems safe to assume that more value-added process business and facilitated network models will eventually emerge.

**Conclusions:**

The overall argument of this article holds important implications for future research: how can policymakers generate adequate leverage to introduce such changes without destroying necessary hospital capacity and the ability to produce quality healthcare.

## Background

Clinically, hospitals still form a cornerstone of health care delivery systems, but important parts of (future) growth are now reserved for community care, outpatient surgery, and other outpatient services [38]. Moreover, while prior technological innovations were once a main factor in pushing doctors into hospitals, current developments now enable physicians to perform an increasing share of their workload outside the bricks and mortar of community hospitals. This movement of care outside hospital walls is being further reinforced by rising numbers of chronic diseases such as obesity and diabetes, which increase the relevance of behavioural not medical strategies. All these extra-hospital forces can be expected to intensify as a result of austerity measures adopted across Europe since the onset of the 2008 financial crisis [43, 49].

Financially, hospitals expend a large part of healthcare’s fiscal appropriations and are a very ‘visible’ part of the healthcare budget. Many countries still enforce a central allocation for large hospital capital projects and most hospital payment schemes retain elements of global budgeting. Hospital expenditure alongside pharmaceutical costs form top priorities for current and additional cuts among budgetary decision makers [33]. However, routine incremental budgeting strategies such as temporary accumulating provider deficits, postponing capital projects, and slicing reimbursement rates across the board cannot provide a viable long-term solution.

Lastly and most relevantly for national health policymakers, when looked at from an organizational perspective, hospitals now form a mature industry, with the number of hospital beds having declined for a considerable period of time [32], and with fiscal pressures on hospitals to make more fundamental changes that nonetheless continue to increase. This unrelenting fiscal pressure also impacts the relationship between medicine and management, as physician-entrepreneurs, be it in their field of expertise or those that are more commercially aware, emerge to help shape the future of the hospital sector [10, 36].

This article examines how changes in hospital structure and funding combine with tightening austerity measures to affect the process and outcome of hospital governance. It explores the fundamental tension between how hospitals have evolved and two main political strategies —payment reform and privatization— which have been advanced thus far to deal with financial austerity as well as the above-noted technological and epidemiological trends that shift healthcare provision away from hospitals. Section 2 describes why payment reform and privatization have become important aspects of policy strategies. Section 3 analyses further pressures on traditional hospital business models. Sections 4 (payment reform) and 5 (privatization) consider the evidence on these topics well as technical and non-technical rationales of moving in these directions. Section 6 covers several important methodological limitations. Section 7 concludes and summarizes the contribution of this paper.

### Maintaining the hospital sector with payment reform and privatization

There exists a large body of literature that describes how hospitals gradually evolved from alms houses and nursing homes towards a combination of what currently are its core assets: emergency departments, operation theatres and (expensive) specialized equipment surrounded by ICU and CCU beds. Medium care nursing units and outpatient departments now are often literally situated as a second circle around these core assets. We do know from a large base of studies and analyses about the formidable veto powers and political influence of hospitals and their doctors [14, 21]. Hospital access was very important for physicians and continual professional conflicts were natural [1, 46].

Such conflicts contributed to the shaping of gatekeeper systems in the UK and the Netherlands among other countries. Gatekeeping helped to guarantee that doctors in- and outside hospitals had their own patient base. The idea that gatekeeping is also a useful precondition for efficiency, which has been adopted by about half of EU health systems [41], only developed later on. In Germany, a line was drawn between all outpatient and all inpatient care, meaning that hospitals were -and to a large extent still are- strictly focused on inpatient care, which is thought by some commentators to have increased the volume of duplicative procedures and diagnostics as well as the length of stay [44]. The professional importance of hospitals was also underlined by the fact that it was quite common in the first half of the twentieth century that (groups of) doctors built private clinics if they did not have access to other hospital ownership types [23]. In countries with substantial numbers of non-profit hospitals such as the Netherlands, Belgium and the populous industrial areas of the Western part of Germany, this was less common. However, a private parallel provider system is still visible in countries where the formalization of universal access is more recent, such as in parts of Southern Europe. The monolithic and underfunded public ownership-led hospital sector in Eastern Europe and the Soviet Union was in essence dismantled as remarkably fast as it was built [28].

During the era of the European welfare state, the increasingly prestigious and ever more expensive hospital sector led to a series of complicated funding issues. Maintenance of assets instead of expansion of capacity came to the fore. To distribute and fine tune existing resources, countries needed payment systems. For hospitals, this usually (but not exclusively) implied separate models between the funding for capital investments and the funding of current or operating costs [23]. In general, public interference was larger for capital appropriations with certificate-of-need regulations. Current costs were overwhelmingly paid on a per diem base, more recently followed by activity-based payment categories such as diagnostic-related-groups. However, such payment models were strictly regulated and the prevalent solutions in Europe remained for decades under the umbrella of the public sector. In search of value-for-money and hospital efficiency, however, hospital management/ownership patterns are now changing and different payment models are being tried.

Two trends have become influential. The first includes a wave of reform in the running of public hospitals, starting with the application of various New Public Management models [20, 34] which transformed public hospitals into various types of semi-autonomous institutions [42] funded by range of different contracting arrangements both inside the public sector and between public and private institutions. With the exception of university clinics, the boundaries in many countries between the public and private sectors have increasingly become blurred [40]. Examples include the creation of foundation trusts in the UK as well as the transformation in the late 1980s in Germany of directly steered public hospitals into independent public companies. There also has been noticeable growth in the size and capacity of the for-profit hospital sector (Table 1).

**Table 1:** For-profit hospital beds

		1995	2000	2005	2010	2013
Austria	Total beds	67.853	63.674	63.248	64.008	56.347
	% for-profit	6,9	7,1	9,0	11,1	15,1
Czech	Total beds	87.784	79.985	77.309	73.746	67.888
	% for-profit				13,7	17.7
Denmark	Total beds		22.927	20.902	19.405	17.241
	% for-profits		0,1	1,4	2,1	2,1
Finland	Total beds	41.483	39.026	37.000	31.395	26.429
	% for-profits	3,3	3,3	3,7	4,4	4,2
France	Total beds		484.279	455.175	416.710	413.206
	% for-profits		19,8	20,4	23,4	23,7
Germany	Total beds			698.303	674.473	667.560
	% for-profits			26,2	29,7	29,8
Italy	Total beds			234.375	215.980	203.723
	% for-profits			28,1	28,0	27,6
Netherlands	Total beds	81.437	76.859	72.698	76.980	
	% for-profit	0	0	0	0	
Poland	Total beds			248.860	251.456	252.281
	% for-profit			17,0	24,3	26,8
Spain	Total beds	154.644	148,081	145.863	145.199	138.153
	% for-profit	19,4	17,9	19,6	17,7	18,8

(Source: OECD health data, January 8th 2016)

In most European countries, private for-profit hospitals have grabbed a bigger share of the market, mostly by acquiring and re-structuring other hospital types. In most countries the private sector also is becoming increasingly consolidated into a few large groups [30]. Hospital groups such as Helios (Germany), Asklepios (Germany), Generale de Sante (France), Capio (Sweden), Quiron (Spain), and the General Healthcare Group and Spire Healthcare (UK) each now own dozens of facilities. One notable impact of this real privatization (e.g. change in ownership to profit-making institutions) has been that the allocation of capital in this part of the health care system has become governed by requirements set by the financial markets. Change from public to private ownership forms an inherent political dispute [46]. In addition to technical arguments regarding efficiency, as for example have been put forward by New Labour when they opened up the NHS to the independent sector at the beginning of this century, ownership changes involve ideological questions in which social norms and values come to the fore [26].

The reform of payment schedules was a second trend and generally is debated in more technical terms if compared to issues of hospital privatization. Hospitals now are typically paid through some kind of activity-based costing system (diagnosis related groups or DRGs) in most OECD countries [45]. The idea was that such a payment model induces shorter average-length-of-stay and thus fosters internal operating efficiency while also reducing waiting lists. Most studies do indicate that DRGs have contributed to lower length-of-stays, but also that they offer few incentives to control overall costs of hospital care [9]. However, in all countries hospital prices are still overwhelmingly fixed by regulation, with the Netherlands as a notable exception. In that country, 70 % of hospital services turnover is freely negotiable; early evaluations indicate that in these free segments prices have come down further, while at the same time volumes have increased more rapidly [22]. More recently, some countries have tried to add pay-for-performance elements to these reimbursement models. The idea was to stimulate the actual benefits per euro spent. The results have been inconclusive; although one recent study from the UK found relative mortality improvements [47], these improvements were not sustained over a longer period [39].

### Pressures on traditional hospital business models

Contemporary European hospitals operate a variety of different business models inside a single organization. For example diagnostics, office visits, elective surgery, emergency treatment or nursing and rehabilitation require different assets, human skills, and organizational procedures. However, since hospitals tend to deliver as many services as possible, these expansionist strategies inevitably increase the complexity of these organizations. Current ‘business school’ strategies for hospital redesign try to untangle such complexities and build more coherent groups of assets, such as focused factories or facilitated network organizations [10, 18, 36].

Calls also exists for a fundamental redirection of the delivery system itself, which requires a paradigm shift and the adoption of patient-centric integrated care, improved hospital efficiency, and interventions in an optimal setting, either in hospitals, at home or in communities’ [13]. Inpatient elective surgeries, once a cornerstone for the use of a hospital building, have started to decline among 14 selected procedures for Medicare beneficiaries [8]. Health care systems across Europe now need to tackle system-wide inefficiencies in the balance of care provided between hospitals, primary care and other settings, particularly after governments have allowed substantial duplication, imbalance and overlap under different justifications [3].

Other than their very substantial size and bargaining power, hospitals hold few strong cards; increasing shares of their activities are performed on an outpatient basis, whether inside or outside the institution. The value of the activities undertaken in hospitals will depend ultimately on (i) the quality of the care provided and the lean implementation of such procedures, and (ii) on the impact of such care on patients’ health in comparative terms with primary care and ambulatory or hybrid modalities. The potential reach of such structural changes might be large; the traditional network advantages of hospital systems seem to decline. Preserving (hospital) organisations is not seen as a sustainable strategy to deliver value [12].

Such statements align with Clayton Christensen’s contention [10] that hospitals still mainly function as solution shops (non-standardized processes to solve complex diagnostic puzzles) and that the autonomization of (part of) the delivered services into value-added process business and facilitated networks are in a preliminary phase. Value-adding process activities are designed to optimize outcomes and reduce variety by standardization of proven techniques. The Martini Clinic in Hamburg is a well-known showcase for its superior outcome in the area of prostate cancer [37]. Facilitated networks depend on optimal organization, facilitation, and operation of networks and have been proven promising in the coordination of chronic illnesses such as Parkinsons’ disease in the Netherlands [5].

Regarding value-added process business: ‘assembly lines’ of low cost, high frequency, low variation services still spread hesitantly in most countries. It is here where the for-profit sector is gaining most ground, although it is also accused of cherry-picking. In contrast to public and non-profit ownership types, for-profits also seem to have much more aggressively relied on traditional network efficiencies by building larger provider groups (back-office integration and standardization). Facilitated network organizations and e-health procedures hold potentially even more disruptive pressures. Such models rely on patient involvement and intensive communication between professionals. However, integrative models of outpatient departments and advanced primary care functions supplemented by new modalities, such as e-health and patient networks, often have difficulties accessing the healthcare markets and obtaining reimbursement due to their non-traditional character where scheduled appointments and treatments in physicians’ offices and clinics might no longer be needed.

Christensen’s solution shop hospital stays important for complex treatments—neonatology, neurosurgery, many cancers etc. Despite their routine complaints about cherry picking by other providers, it is here where tertiary hospitals continue to have substantial leverage. ‘Palaces of complexity’ that handle high cost, high variation, and low frequency care are still needed in the distant and not so distant future. From that perspective, it is the less complex European community hospital that is under threat due to the growth of modern outpatient centers and facilitated networks, not the large academic providers such as Karolinska in Stockholm, Charité Hospitals in Berlin, or Guy’s and St. Thomas in London. Nevertheless, further growth of these new decentralized and often private provider models will necessarily change the relation between medicine and management. Other skills that relate to ICT and competencies of operational excellence will increase in relevance versus those that are needed for routine medical procedures and general consults at the doctor’s office.

### Payment reform: ‘savings’ and ‘more quality’?

Hospitals need payment schemes. Such schemes may focus on beds, hospital stays, physician fees, global budgets, specific procedures and treatments, outlier payments and add-ons for certain expensive pharmaceuticals and treatments, or diagnostic-related payment. Modern healthcare systems register increasing amounts of data and more and more seek to pay for quality of care as the prime proxy for outcome of hospital treatments. Payment reform is framed as simply a technical effort to pay (higher amounts) for better outcomes and quality of care. Many countries are experimenting with paying for performance (especially in primary care), paying for the entire continuum of care (bundling), and paying capitated models.

Substantial numbers of policymakers hold high hopes that paying for quality will eventually solve adverse effects of the earlier reimbursement schemes. However, a number of fundamental problems still need to be resolved, such as the possible crowding out of intrinsic motivation and the use of rewards versus penalties [11]. If these payment reforms seek cost-control, as many state they do, complicated design issues grow in political importance. Cost-containment is not a primary goal for all stakeholders. Hospitals will typically marshall fierce opposition towards unwelcome forms of cost-control and harness politicians to their defense who feel that the European welfare state has come under too much pressure. Another (neglected) difficulty might be that in such efforts of payment reform, modern ICT possibilities can very well increase the coordination possibilities of agents and local actors versus their principals.

#### Pay for performance

‘Best-practice prices’ and ‘pay for performance’ (P4P) mechanisms of hospital care do require detailed information on input mixes, but also on quality, as well as on medical evidence and clinical agreement concerning what constitutes good practice both inside and outside the hospital—about which information is even more scarce [7]. It is far from clear that European health systems will produce any of these data trails sufficiently soon given the current level of development of health technology assessment (HTA) and of clinical registries. Regarding the latter, Sweden’s quality registries do form an exception but the effective use of such tools continues to be a challenge. Physician-led efforts to reduce unnecessary tests, treatments and procedures such as the international spread of the US ‘Choosing Wisely’ campaign also do not see visible roles for payment systems and purchasers that might unintentionally harm patients [25]. In the UK the high expectations of the Quality and Outcomes Framework for primary care, perhaps the largest P4P experiments in Europe, have so far not been met [15].

#### Bundling

Another pressing demand will be the delivery of better-coordinated care for chronic disease patients. Reforming the structure of payment systems towards bundled payments has been one answer, although with inconclusive outcomes so far [27]. One example is the funding of diabetes in the Netherlands; a first evaluation shows that overall quality of care did improve (although a control group was not included) but hospitals recouped any losses with additional charges for high-risk diabetes patients, suggesting up-coding [6]. This illustrates that the development of bundled payments at least from the perspective of cost-containment will be complex (and is prone to the influence of interest groups). One underlying characteristic that induces adverse outcomes might be the strict organizational separation between hospital care and primary and ambulatory care sanctioned by legislation, with hospital staff as salaried employees of local or state government while staff outside hospitals are more often private, organised in small groups.

#### Capitation and shared-savings

Models that combine risk-adjusted capitation, shared-savings and incentives for quality of care, such as the Alternative Quality Contract in Massachusetts and the Accountable Care Organization in Medicare, are hailed as the next solution that will combine cost control and better quality of care, typically with inclusion of indicators to improve population value (triple aim). Value-based contracting with weighted capitation funding defining ‘blocks’ of services—such as maternity, acute trauma care, care for the frail elderly and chronic care—that would stretch across a number of care settings and providers is an important challenge.

Europe holds some experiences of its own with these payment innovations. *Gesundes Kinzigtal*, a small-scale experiment in southwest Germany claims substantial savings along improvements in quality of care [19]. Capitated funding models jointly for hospital and primary care is also currently the case in Alzira/ Valencia, Spain. Despite theoretical attractions, tremendous practical challenges remain such as accountability and related regulations, cost and quality metrics, information technology, clinical governance and the development of a shared organisational culture. In this last regard, it is unclear at this stage to what extent the evaluation of Alzira is really known, as data published are rather incomplete [2]. It is also important to note that although some high profile studies do show favourable trends of these models [31, 51], these savings do not necessarily or automatically accrue to patients or tax payers; physicians and to a lesser extent purchasers seem to benefit the most from increasing margins, which in Europe might come with questions about the appropriateness of such consequences.

### Hospital privatization: ‘stakeholders’ or ‘shareholders’

Hospital management in much of Europe typically decides on internal professional structures, on hiring and firing, on performance-related incentives, on day-to-day activity monitoring, on carrying out data collection for national/regional governments and other payers and stakeholders, on criteria to evaluate whether objectives have been achieved and on performance indicators (irrespective of whether or not they are later published). However, especially in public hospitals, politicians (directly by sitting on the boards or through appointed board members) continue to play a major strategic role: they set objectives, establish operational boundaries through planning and staffing-level requirements, negotiate and regulate staffing contracts and payment levels, and make final decisions about financial dependence. Hospitals are major local employers and thus politicians often have a firm electoral interest in the continuation of current activity and employment levels at these institutions. Not surprisingly, they tend to be hesitant to initiate major organizational change, usually preferring to add new layers of health care institutions crystallized under very different forms and functions in times of budgetary expansion. On the other hand, indebted public hospitals form large liabilities on public balance sheets.

#### Privatization

Some political actors view hospital privatisation (with its correlates of for-profit-making full autonomy, market segmentation and product specialization) as an attractive option to handle this list of problems. Such discussions however come with strong non-technical and political rationales [46] and cannot be seen as entirely separate from broader developments to retrench and redirect the European welfare state. Recent trends in some tax-based European countries suggest that some senior political figures are willing to experiment with various forms of changed ownership in an effort to produce better clinical and financial results (see Table 1). The existing evidence about the growth of private hospitals in some social insurance based countries has attracted considerable attention. The private sector now operates more than one in every four hospital beds in Germany and one in every four in France [30]. In England, which has two decades of sometimes rocky experimentation with changed hospital ownership structures, commissioning of independent sector providers by the English NHS recently accounted for 3.5 % of NHS-funded first outpatient appointments, however this percentage is substantially higher for certain elective procedures such as hip replacements and gallbladder removals [24]. On the negative side, the first privately run NHS hospital failed after a critical report on its quality and funding pressures. Circle, its mother company, pulled the plug from the deal (the Guardian, January 9th 2015). It remains important to note that on top of their growing role in tax-funded health care delivery systems, for-profit providers continue to serve as a parallel system for well-off inhabitants in Central and Southern Europe as well as in England.

What do we actually know about the technical performance of private for-profit hospitals? In Europe, public, non-profit, and for-profit hospitals often co-exist. Economic theory tells us that enterprise models are more efficient if formal ownership controls equals effective control (thus there are no, or few, agency problems), and will attract for-profit entrants if decent returns on investment are probable. Hospital margins depend on high prices (e.g. due to market power and lack of price sensitivity) as well as on low costs (due to higher efficiency as well as access to scarce production factors, such as capital and physicians). Kenneth Arrow pointed towards information asymmetry as an important market failure and a main rationale for the dominance of non-profit (and public) hospitals above for-profit competitors in well-functioning hospital markets. Others such as Pauly and Redisch [35] contend that non-profit hospital providers hurt social efficiency because they tend to act in the interests of the self-employed physicians, if such are ‘non-contractable’. Many economists hold the opinion that if services become more ‘contractable’, the rationale for for-profit ownership increases.

An account of the efficiency of for-profit hospitals still relies largely on peer-reviewed US studies. Systematic meta-reviews point towards the fact that for-profit hospitals generally (i) provide less community benefits, (ii) have somewhat worse mortality ratios in comparison with non-profit providers but better than public hospitals, and (iii) have few differences in cost-efficiency in comparison with other ownership types. Most studies illustrate that differences within hospital ownership types are far more important than differences between for-profits and public or non-profit hospitals [23].

However, such sobering results come with caveats from a European perspective. The fact that for-profits provide less uncompensated care is less relevant in European countries that generally reimburse all necessary hospital services. Longitudinal studies can also come to other conclusions than cross-sectional research. Herr [17] points to the fact that even if (German) for-profits are less efficient, they may actually increase efficiency if they acquire inefficient public or non-profit hospitals, a major strategy for their growth. For-profits generally are more responsive to (financial) incentives than other ownership types [16]. Some studies also showed somewhat better performance on quality indicators for French [29] and German for-profits [48]. Another recent study indicated that in France elective admissions to for-profit providers are shorter as well as less expensive, compared to other provider types [50].

Thus most technical evidence on the efficiency of for-profit ownership seems to be inconclusive. But what about the ‘winners’ and ‘losers’ of for-profit hospital margins and cost structures versus other hospital types? A consistent picture exists among the available evidence, both in the US as well as Europe [23]. On top of a comparable cost-base, for-profits do calculate a higher margin, which rewards ‘shareholders’ but typically these funds come from savings in expenses paid to other ‘stakeholders’ such as tax-payers and insurers. For-profits do appear to have a different cost-structure with higher compensation for both management and medical staff, but lower wages for nurses and other employee categories. As with payment reforms, privatization comes with important political disputes. Non-technical changes in the distribution of resources and rents might very well be much more important than any technical changes on costs or quality of care. However, for-profits are more responsive to incentives and other changes and over time may well contribute towards strategies that seek to dismantle the traditional hospital with business models that support value-adding process activities and facilitated networks.

### Limitations

This review necessarily has several methodological caveats and limitations. It does not describe in depth the wide range of external factors that may be shaping the future development of the hospital sector. An increasingly international healthcare workforce market and different workforce planning assumptions in nation states could impact hospitals’ ability to operate efficiently. If occupancy rates are currently above 85 %, as they are in many European hospitals, declining occupancy rates might also improve bed availability in emergency cases and thus medical outcomes [4]. Due to larger numbers of immigrants and refugees, hospitals may increasingly act as a safety net for basic services in a number of countries. However, these potential complications do not undercut the article’s main argument that hospitals face increasing difficulties in maintaining their current business models.

There are other methodological limitations that should be noted as well. Hospitals are not always comparable institutions and focus on different competencies and specializations, running from small community hospitals to large university clinics. Publicly operated hospitals can vary considerably from directly managed units through to near-independently-managed entities. The range of hospital ownership arrangements across Europe varies from publicly owned (national, regional or municipal) to various types of private not-for-profit (religious and/or voluntary) or private for-profit companies [40]. As a consequence of this great diversity, observations made in this article are broadly summatory in nature, and before application would need to be passed through the prism of each country’s specific institutional structures as well as the social norms and values that guide policy decision-making within it.

## Conclusions

This article examines the uncomfortable realities that the European hospital sector currently faces and the potential impact of wide-spread rationalization policies such as (hospital) payment reform and privatization. Based on the evidence we present, such policies will probably fall short in delivering better quality of care and lower growth in health expenses. Reasons can be sought in a mix of evidence on the effectiveness of these rationalization policies, which probably will reflect important non-technical and political rationales that in practice pursue other goals, such as protecting vested interests of the hospitals as well as many difficult-to-handle technical complexities that guide these policy instruments.

The above conclusions suggest decidedly modest expectations for the solutions proposed by business school professors such as Michael Porter and Clayton Christensen. To be sure, medical professions’ top performers will continue to need academic and tertiary care hospitals for their solution shop as they seek to explore important new frontiers in patient care. Nevertheless, pressures for different business models will gradually continue to increase and it seems safe to assume that more value-added process business and facilitated network models will eventually emerge, although it is uncertain when such developments will disrupt the business model of the hospital as we know it today. Our overall argument holds important implications for future research: how can policymakers generate adequate leverage to introduce such changes without destroying necessary hospital capacity and the ability to produce quality healthcare?

Up to a certain extent, European countries do seem receptive towards hospital consolidation and privatization. In many countries, private hospital groups are at the forefront of this consolidation process to build national or even pan-national networks of hospitals. Privatization might increase the responsiveness of healthcare systems, especially of ‘contractable’ services and improve the performance of the worst providers, if acquired. These might be important prerequisites for necessary changes. However privatization does not seem to provide an answer to the need for savings simply for being too volume- and price- driven, although short-term operating budget savings might occur due to the selling of public assets (often below their long-term market value).

Policy learning is needed for real change that can tackle the challenges which cannot be solved within the current hospital landscape and current relations between medicine and management. Europe forms an ongoing natural laboratory test that melts distinctions between political, managerial and administrative realms as well as between policy formulation and implementation in a crowded arena. Both in the public and private health sectors, hospitals are increasingly seen as transitory stepping stones with blurred boundaries, but this transition is still unclear at the moment in terms of destination. Most countries do not have active purchasers of care with the capacity to make refined, value-based contracting arrangements with providers. Only when the purchasing function in European healthcare has been strengthened will the patterns of change in hospital care show identifiable trends.

In the meantime, outcomes from the present combination of organizational pressures and financial restriction will as always differ in line with normative, organizational and cultural issues. Similar solutions may well work out differently across Europe due to these contextual differences. A general pattern however can perhaps be expected: policymakers and public budget holders will continue to face a tough time as they try to respond to being boxed in by hospital spending on the one hand and revenue austerity on the other. Such challenges used to be handled by top-down freezing of budgets, although with few directions about where to seek efficiency savings. More of both might be needed.
